# Integration analysis of MKK and MAPK family members highlights potential MAPK signaling modules in cotton

**DOI:** 10.1038/srep29781

**Published:** 2016-07-15

**Authors:** Xueying Zhang, Xiaoyang Xu, Yujia Yu, Chuan Chen, Jing Wang, Caiping Cai, Wangzhen Guo

**Affiliations:** 1 State Key Laboratory of Crop Genetics & Germplasm Enhancement, Hybrid Cotton R&D Engineering Research Center, Ministry of Education, Nanjing Agricultural University, Nanjing 210095, China

## Abstract

Mitogen-activated protein kinase (MAPK) cascades play a crucial role in plant growth and development, as well as their biotic and abiotic stress responses. As a nodal point of the MAPK cascade, the MKK gene family has not been systematically studied in cotton. Here, we identified 11 putative MKK genes in the *Gossypium raimondii* genome. Phylogenetic analysis showed that the MKKs were supported by architectures of conserved protein motifs. Expression patterns of MKKs under hormone treatments or abiotic stresses revealed their diverse functions in stress responses. Based on a yeast two hybrid, a total of 63 interactive pairs of MKKs and MAPKs were identified in cotton. Among these, 40 interactive pairs were newly identified compared to that reported previously in *Arabidopsis*. Integration analysis of the interaction network and expression patterns of MKK and MAPK family members revealed 13 potential MAPK signaling modules that are involved in the complicated cross-talk between hormones and abiotic stresses. Taken together, our data enhance the understanding of the evolution and function of MAPK cascades in cotton, and lay the foundation for the improvement of various defense responses that use MAPK signaling modules in the future.

Mitogen-activated protein kinase (MAPK) cascades are evolutionarily conserved and fundamental signal transduction pathways that play roles upstream of various receptors/sensors that transduce extracellular stimuli into intracellular responses in eukaryotes. A canonical MAPK cascade consists of three functionally linked protein kinases. MAPKs are phosphorylated at their threonine and tyrosine (TXY) residues and are activated by a MAPK kinase (MAPKK), which itself is phosphorylated at its serine/threonine residues in the S/T motif and is activated by MAPKK kinase (MAPKKK). Numerous reports have provided evidence that the MAPK cascades play pivotal roles in the transduction of diverse cellular processes, such as salinity stress, drought, temperature, wounding, pathogen attack and plant hormone responses^1^,^2^,^3^.

Many members of MAPK cascades have been identified in a variety of plants after the completion of the whole genome sequence. A total of 80 MAPKKKs, 10 MAPKKs and 20 MAPKs have been characterized in the *Arabidopsis* genome^4^,^5^; 75 MAPKKKs, 8 MAPKKs and 17 MAPKs are present in the *O. sativa* genome^6^,^7^; 89 MAPKKKs, 6 MAPKKs and 16 MAPKs have been reported in tomato^8^,^9^; 74 putative MAPKKKs, 9 MAPKKs and 19 MAPKs have been found in maize^10^,^11^; 59 MAPKKKs, 6 MAPKKs and 14 MAPKs have recently been identified in cucumber^12^; and 75 MAPKKKs, 12 MAPKKs and 16 MAPKs are present in the *B. distachyon* genome^13^. To date, few MAPK signaling cascades have been characterized in *Arabidopsis*. MEKK1 is active upstream of MKK1 and MPK4 in flagellin and ROS signaling^14^,^15^, and also plays a role upstream of MKK2 and MPK4, which can be activated during cold acclimation and contributes to the acquisition of freezing tolerance^16^. The MEKK1-MKK1/2-MPK4 cascade was shown to positively regulate defense responses against necrotrophic fungi while negatively regulating defenses against biotrophic pathogens^17^. MKK1/2-MPK4/6 cascades have been shown to play important roles in the response to salt and cold stress, as well as pathogen infection^18^,^19^. MKK3 displays an atypical MKK structure, which can act on MPK7 and MPK8 to mediate ROS signaling and regulate MPK6 in response to jasmonic acid^20^,^21^. In addition, the MEKK17/18-MKK3-MPK1/2/7/14 cascade is an ABA dependent MAPK pathway and plays a role in ABA stress signaling^22^. MKK4/5-MPK3/6 cascades play vital roles in plant innate immunity^23^,^24^, and the MKK1-MPK3/6 and MKK4/5-MPK3/6 modules participate in stomatal development and stomatal dynamics^25^,^26^. MKK6-MPK4/11 cascades directly regulate cytokinesis and mitosis^27^, while MKK9-MPK3/6 cascades regulate ethylene signaling and camalexin biosynthesis, and may also play a role in leaf senescence^28^,^29^.

Cotton is one of the most important economic crops in the world, and provides the world’s leading natural textile fiber and considerable amounts of edible oil. Several MKKs have been reported in cotton. *MKK1* is responsive to salt and drought stress: Ectopic expression of *MKK1* in *N. benthamiana* plants enhanced their salt and drought tolerance, but increased the transgenic plants’ pathogen sensitivity^30^. *MKK2* plays an important role in pathogen attack and in silencing *MKK2*-compromised cotton resistance to *Verticillium wilt*^31^. *MKK4* plays an important role in abscisic acid-induced catalase1 expression and H_2_O_2_ production^32^, while *MKK5* can be induced by pathogen infection, abiotic stresses and multiple defense-related signaling molecules. Overexpression of *MKK5* in *N. benthamiana* enhanced the plants’ resistance to bacterial pathogens, but reduced their tolerance to salt and drought stresses^33^. A growing body of evidence suggests that there are fewer MKKs than MAPKKKs and MAPKs, suggesting that individual MKKs may function as bifurcation points and are likely to be involved in multiple MAPK cascades in response to a variety of stresses^34^. In addition to those described above, a total of 78 MAPKKK genes and 28 MAPK genes have been identified in the *G. raimondii* genome^35^,^36^. In spite of this, the systematical investigation of the MKK gene families in cotton and the verification of the interactions between MKK and MAPK members in MAPK cascades have not been studied. Here, we identified 11 MKK genes in the *G. raimondii* genome through database searches, and classified them according to their homology with those in *Arabidopsis*. We analyzed their chromosomal location, sequence phylogeny, genomic structure, and evolutionary mechanisms. We subsequently, investigated the expression level of MKK genes in different tissues and in response to different hormone, temperature and stress treatments in upland cotton. Furthermore, we cloned their corresponding orthologous MKK genes in *G. hirsutum*, examined the interaction profile of MKK-MAPK proteins using yeast two-hybrid (Y2H) assays, and compared the interaction network and expression patterns of MKKs and MAPKs, which showed that several potential MAPK signaling modules were involved in the complicated cross-talk between hormones and abiotic stresses. Through this work, we can better understand signal-related stress and abiotic stress responses, as well as the corresponding molecular mechanisms regulated by MAPK cascades, in cotton. The data presented here lay a solid foundation for a deeper understanding of MAPK cascades and for future improvement of various defense responses using the signaling pathways in cotton breeding.

## Results

### Identification of MKK Genes in Cotton

**Figure 1:**
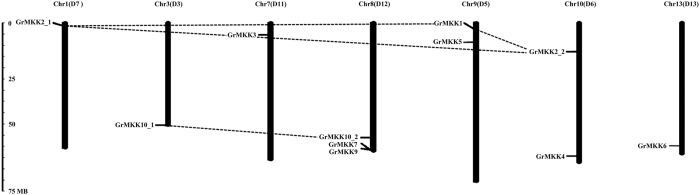
Chromosomal distribution of MKK genes in *G. raimondii*. Eleven candidate MKK genes were matched to 7 chromosome of the *G. raimondii* genome^37^. The nomenclature of MKK genes in *G. raimondii* were based on MKKs in *A*. *thaliana*. Lines were drawn to connect duplicated genes.

To identify MKK genes in *G. raimondii*^37^, we used 18 MAPKK protein sequences, 10 from *Arabidopsis* and 8 from *O. sativa*, as direct queries to screen the *G. raimondii* genomic database (http://www.phytozome.net). As a result, a total of 11 MKK genes were identified in *G. raimondii*. With the release of whole-genome sequence information of allotetraploid *G. hirsutum* (Gh)^38^ and *G. barbadense* (Gb)^39^, and diploid *G. arboreum* (Ga)^40^, we further identified 20, 20 and 11 MKK genes from the three cotton species, respectively (Supplementary Dataset 1). Notably, the number of MKK genes in allotetraploid cotton is twice as many as in diploid cotton. Nine genes had one copy in the diploid *G. raimondii* and *G. arboreum* genomes, and two homologous genes (homoeologs from the A and D subgenomes) were found in the tetraploid *G. hirsutum* and *G. barbadense* genomes. The homoeologs of two were inconsistent, which might result from gene loss during their individual evolutionary processes or from assembly error in partial chromosomal regions, and needs to be further confirmed. These MKK genes were predicted to encode proteins from 320 to 518 amino acids in length, with putative molecular weights (MWs) ranging from 35.42 to 57.65 KDa and isoelectric points (pIs) ranging from 5.49 to 8.92 (Table 1). To elucidate the chromosomal distribution of these MKK genes, we integrated 13 scaffolds of the *G. raimondii* genome^37^ (named as Chr. 1 to Chr. 13) with the reported high-density interspecific genetic map of allotetraploid cultivated cotton species, and confirmed that the 11 candidate MKK genes matched to 7 chromosomes (Fig. 1). Each gene was named according its amino acid homology with that in *Arabidopsis*, and if two or more genes had the same homologs in *Arabidopsis*, they were distinguished by an extra number. Information on the MKK genes in *G. raimondii*, including their names, origins, chromosome locations, pIs, MWs and subcellular localizations, are shown in Table 1.

**Figure 2:**
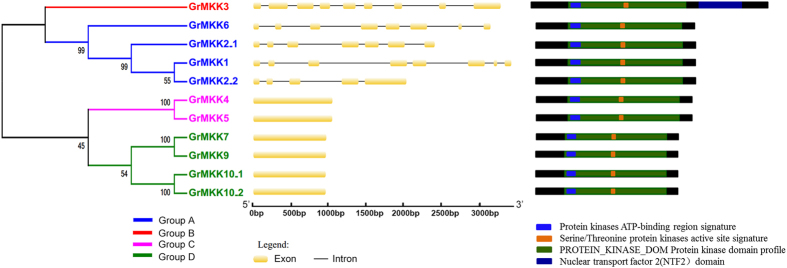
Phylogenetic analysis, intron-exon identification and structural comparison of 11 MKK genes in *G. raimondii*. A phylogenetic tree was constructed using MEGA 6 by ML method with 1000 bootstrap replicates. Intron and exon were represented by black line and orange box. Four conserved domains of each MKK gene were shown with blue, orange, green and dark blue, respectively.

The *G. raimondii* genome underwent at least two rounds of genome-wide duplication^37^. To understand the mechanism of expansion of the 11 MKK genes in *G. raimondii*, we investigated tandem and segmental duplication events of MKK gene family members on 7 chromosomes by genome synteny analysis. As shown in Fig. 1, five paralogs of 11 *GrMKKs* were identified, including four segmental duplications (*GrMKK1*-*GrMKK2_1*, *GrMKK1*-*GrMKK2_2*, *GrMKK2_1*-*GrMKK2_2* and *GrMKK10_1*-*GrMKK10_2*) and one tandem duplication (*GrMKK7*-*GrMKK9*). Furthermore, these paralogs were clustered together in the phylogenic tree and shared similar exon-intron structures (Fig. 2). These results indicate that segmental duplication events played significant roles in MKK gene expansion in the *G. raimondii* genome.

### Multiple Alignment, Phylogenetic and Domain Analyses of GrMKKs

Alignment of amino acid sequences revealed that the GrMKKs contain 11 domains (I–XI; Supplementary Fig. 1). S/T-X5-S/T motifs in GrMKKs were located in the activation loop between the kinase subdomains VII and VIII. Phylogenetic analysis indicated that the GrMKKs could be divided into four major groups, with four members in group A, four in group D, two in group C, and only GrMKK3 in group B. This is consistent with the phylogenetic relationship of MKKs in *Arabidopsis* (Fig. 2). Analysis of the exon positions and intron phases in MKK genes showed that the members in groups C and D had no introns and only *GrMKK3* in group B contained nine exons. Compared with those in groups B, C and D, the MKKs in group A had a complex distribution of exons and introns, where *GrMKK1* and *GrMKK6* had eight exons, and *GrMKK2_1* and *GrMKK2_2* had seven and five exons, respectively. The diversity in the exon-intron structures among the different phylogenetic subgroups showed that duplication events were likely to have occurred historically, and the offspring genes evolved to have diverse exon-intron structures to accomplish different functions. Conserved domain analysis showed that all the GrMKKs contained three types of special subdomains; the Serine/Threonine protein kinases active site, an ATP binding site and a protein kinase domain. Moreover, GrMKK3 had a unique C-terminal NTF2-like domain, which may be essential for its nuclear localization (Fig. 2).

**Figure 3:**
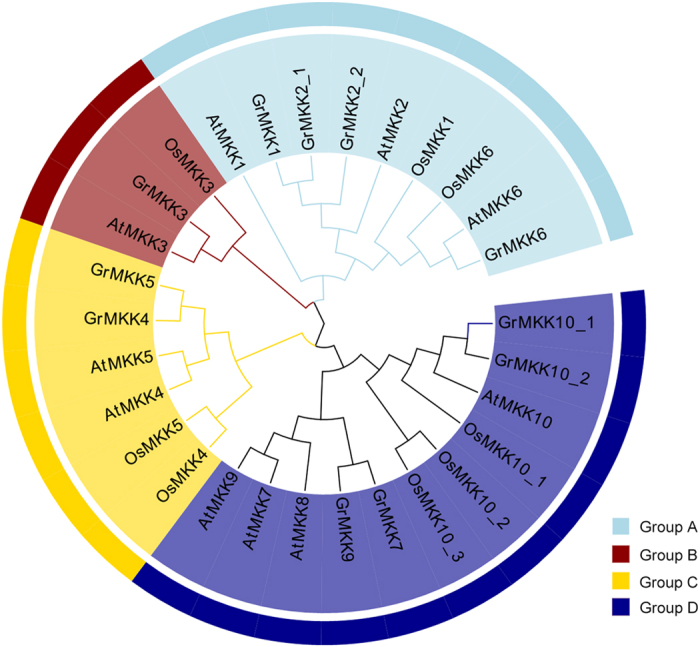
Phylogenetic tree of MKK family genes from *G. raimondii*, *A. thaliana* and *O. sativa*. Amino acid sequences were aligned using Clustal W in MEGA 6 software and ML method was used to perform phylogenetic analysis with 1,000 bootstrap replicates.

To further explore the evolutionary relationships between the MKKs in *G. raimondii* and those in other species, an un-rooted tree was constructed using 10 MKKs from *Arabidopsis*, 8 MKKs from *O. sativa* and 11 MKKs from *G. raimondii*. Phylogenetic analysis indicated that all of the MKKs from different species could be classified into the A, B, C and D groups (Fig. 3). No members of groups B and C, which contained MKK3, MKK4 and MKK5, were missing from the three species; In group A, there were nine MKKs, MKK1, MKK2 and MKK6 in *Arabidopsis*, MKK1, MKK6 in *O. sativa*, and MKK1, MKK2_1, MKK2_2 and MKK6 in *G. raimondii*; In Group D eleven MKKs, MKK7, MKK8, MKK9 and MKK10 in *Arabidopsis*, MKK10_1, MKK10_2, MKK10_3 in *O. sativa* and MKK7, MKK9, MKK10_1 and MKK10_2 in *G. raimondii*, were clustered together. Based on the phylogenetic analysis, MKK family members were conserved among dicots, however some in groups A and D were lost after the divergence of the monocots and dicots. As expected, most of the MKKs in *G. raimondii* were clustered more closely with those in *Arabidopsis* than *O. sativa*.

### Spatial and Temporal Expression of MKK Genes in *G. Hirsutum* TM-1

Cultivated tetraploid cotton species differ greatly with respect to plant morphology as well as economic characteristics, including fiber production, oil content and stresses resistance. To explore the organ-specificity of MKK family members, we examined the transcripts abundance in different tissues of *G. hirsutum* TM-1, including roots, stems, leaves, petals, anthers, ovules and fibers, at three different developmental stages (0 days post-anthesis [dpa], 10 dpa and 20 dpa). As shown in Supplementary Fig. 2, *GhMKK1* and *GhMKK2_2* in group A, *GhMKK3* in group B, and *GhMKK5* in group C were predominantly expressed in vegetative and reproductive organs, with the highest expression observed for *GhMKK2_2* and *GhMKK3* in all tissues and organs. *GhMKK7* in group D was predominantly expressed in vegetative organs and had the highest expression levels in root and leaf tissues. *GhMKK2_1* and *GhMKK6* in group A were predominantly expressed in fibers at different development stages: *GhMKK2_1* was most highly expressed in 20 dpa fibers, while *GhMKK6* was preferentially expressed in 0 dpa ovules. *GhMKK4* in group C was ubiquitously expressed in all tissues but was preferentially expressed in petals. *GhMKK9*, *GhMKK10_1* and *GhMKK10_2* in group D had very low or undetectable expression levels in all tested tissues. MKKs in the same group with high structure identity had diverse expression patterns in different tissues, indicating the functional diversity of duplicated genes. In contrast, however, the similar expression profile of genes from different groups suggested that these paralogs share similar functions.

### Induced Expression Patterns of MKK Genes Under Multiple Phytohormone Treatments and Abiotic Stresses

**Figure 4:**
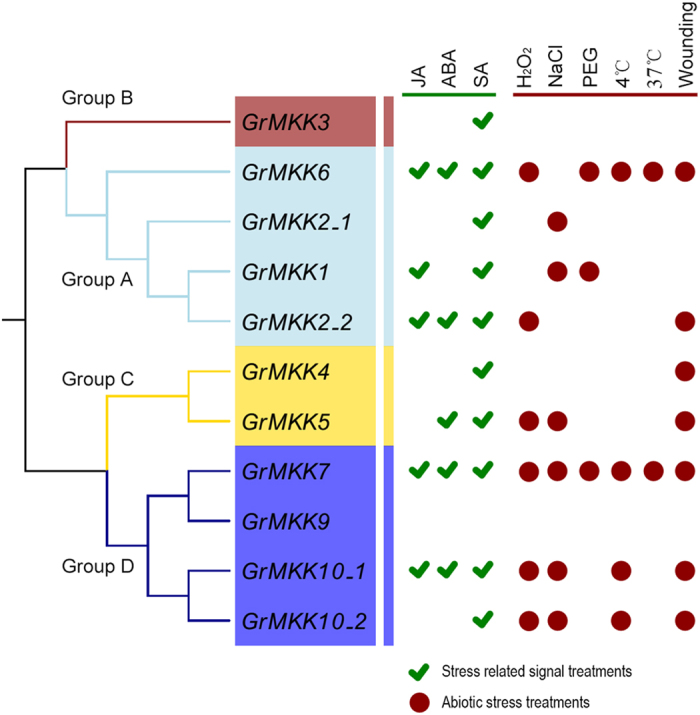
Expression pattern of MKK genes under signal and stress treatment. The tick (green color) indicated the MKKs induced under stress-related signal treatments, including JA, ABA and SA. The circle (dark red) indicated the MKKs induced under stresses treatment, including H_2_O_2_, NaCl, PEG, 4 °C, 37 °C and wounding.

MAPK cascades were not only involved in plant growth and development, but also played key roles in the control of plant responses to multiple environmental stimuli, including abiotic phytohormones and stresses. Here, we conducted qRT-PCR analyses to examine the expression levels of the MKK genes in response to three phytohormones (jasmonic acid [JA], abscisic acid [ABA] and salicylic acid [SA]). With the exception of *MKK9*, the induced expression of all MKKs was detected (Fig. 4). After JA treatment, three MKK genes (*GhMKK1*, *GhMKK2_2* and *GhMKK6*) in group A and two MKK genes (*GhMKK7* and *GhMKK10_1*) in group D were up-regulated, reaching peaks at different time points. In addition, five MKK genes were significantly up-regulated after ABA treatment. Of them, the expression of *GhMKK6* in group A and *GhMKK10_1* in reached a peak at 1 h, and *GhMKK2_2* in group A, *GhMKK5* in group C and *GhMKK7* in group D, reached peaks at 6 or 8 h after treatment. Finally, *GhMKK6* was induced and reached an expression peak at 24 h, and all other MKK genes were significantly up-regulated and reached expression peaks 6 h after SA treatment. Detailed information on the expression patterns of each MKK under each treatment is shown in Supplementary Fig. 3.

The expression patterns of MKK genes following abiotic stress treatment were also analyzed in detail. We performed qRT-PCR to detect differences in their expression after six stress treatments (oxidative stress [H_2_O_2_], salinity, drought, cold, heat and wounding) (Fig. 4). As a result, six MKK genes, two (*GhMKK2_2* and *GhMKK6*) in group A, *GhMKK5* in group C, and three (*GhMKK7*, *GhMKK10_1* and *GhMKK10_2*) in group D, were significantly up-regulated after H_2_O_2_ treatment, and had diverse expression patterns. Also, six MKK genes, *GhMKK1* and *GhMKK2_1* in group A, *GhMKK5* in group C, *GhMKK7*, and *GhMKK10_1* and *GhMKK10_2* in group D, were induced after NaCl treatment and had diverse expression patterns. Three MKK genes were induced by drought treatment; two (*GhMKK1* and *GhMKK6*) in group A and *GhMKK7* in group D. *GhMKK7* was particularly highly induced and reached a peak at 6 h. In addition, after low temperature treatment (4 °C), *GhMKK10_2* was induced and reached a peak at 4 h, and three other MKK genes (*GhMKK6*, *GhMKK7* and *GhMKK10_1*) were induced and highly expressed after 12 h. Moreover, only two MKK genes were induced and highly expressed upon exposure to high temperature conditions (37 °C): *GhMKK6* was induced and reached a peak at 8 h, while *GhMKK7* was up-regulated and highly expressed at 4 and 24 h. Finally, seven of ten MKK genes were induced and reached peaks at 2 h or 6 h when the seedling leaves were cut with scissors; three in group D (*GhMKK7*, *GhMKK10_1* and *GhMKK10_2*), two in group A (*GhMKK6* and *GhMKK2_2*) and two in group C (*GhMKK4* and *GhMKK5*). Further details of the expression patterns of individual genes under each treatment are shown in Supplementary Fig. 4.

Ten MKK genes were responsive to three hormone treatments. *GhMKK2_2*, *GhMKK6*, *GhMKK7* and *GhMKK10_1* were simultaneously induced and accumulated at high levels after all three hormone treatments. *GhMKK1* and *GhMKK5* were induced by two inducers, and *GhMKK2_1*, *GhMKK3, GhMKK4* and *GhMKK10_2* were only induced by SA treatment. All MKK genes, with the exception of *GhMKK3*, were induced by at least one of six stress treatments. *GhMKK4* were only induced by wounding, *GhMKK2_1* was induced by NaCl, and *GhMKK1* and *GhMKK5* were induced by two or three abiotic stress treatments. *GhMKK10_1, GhMKK10_2* and *GhMKK6* were induced by four or five abiotic stress treatments. *GhMKK7* was induced and expressed at high levels under six abiotic stress treatments (Table 2). These expression patterns suggest that MKKs respond to various hormones and carry out multiple physiological functions to help plants adapt to a variety of complex environmental challenges.

To investigate whether these duplicated paralog pairs were with the same expression patterns, we compared the expression profiles of the duplicated paralogs in different organs and under different stress treatments (Supplementary Table 1). There was no significant correlation between the expression of paralogs in different tissues and organs. However, the four paralogs showed the clear positive correlations in ABA, H_2_O_2_ and SA stress-related signals, and *GhMKK2_1* and *GhMKK2_2* were positively correlated following JA treatment. Further, *GhMKK1* was positively correlated with *GhMKK2_2*, and *GhMKK2_1* was positively correlated with *GhMKK2_2* under five abiotic stresses, *GhMKK1* and *GhMKK2_1* were positively correlated under three abiotic stresses (4 °C, 37 °C, wounding), and *GhMKK10_1* and *GhMKK10_2* were positively correlated under NaCl and 37 °C treatments. These results indicate that the duplicated MKK gene pairs had similar functional responses to different stresses, although their expression differed in different tissues and organs.

### Interactions Between MKK and MAPK Protein Family Members

Since the nature of protein kinase activity depends on their direct physical encounters, we conducted a comprehensive directed yeast two-hybrid (Y2H) assay to define the interactions between the last two kinases in the MAPK cascade in cotton. Besides the 28 MAPK genes reported previously^36^, we further confirmed 56, 52 and 28 MAPK genes from *G. hirsutum*, *G. barbadense* and *G. arboreum*, respectively (Supplementary Dataset 2). Due to the high similarity (more than 97%) in the amino acid sequences (Supplementary Table 2) of MKK and MAPK homoeologs in the A- and D-subgenomes, we performed PCR-based cloning for anyone of the MKK homoeologs. In total, eight MKK genes were cloned and sequenced to confirm their complete open reading frame (GenBank accession numbers: KX118695-KX118702) (Table 1). Combined with the 21 MAPK genes cloned previously^36^, we constructed eight MKKs and 21 MAPKs into DNA binding domain and activation domain plasmid vectors, respectively. Each MKK protein was found to have a distinct auto-activation activity in yeast. To solve this issue, Aureobasidin A (ABA) was added to selective medium and the auto-activation of MKKs was inhibited completely (Supplementary Fig. 5).

**Figure 5:**
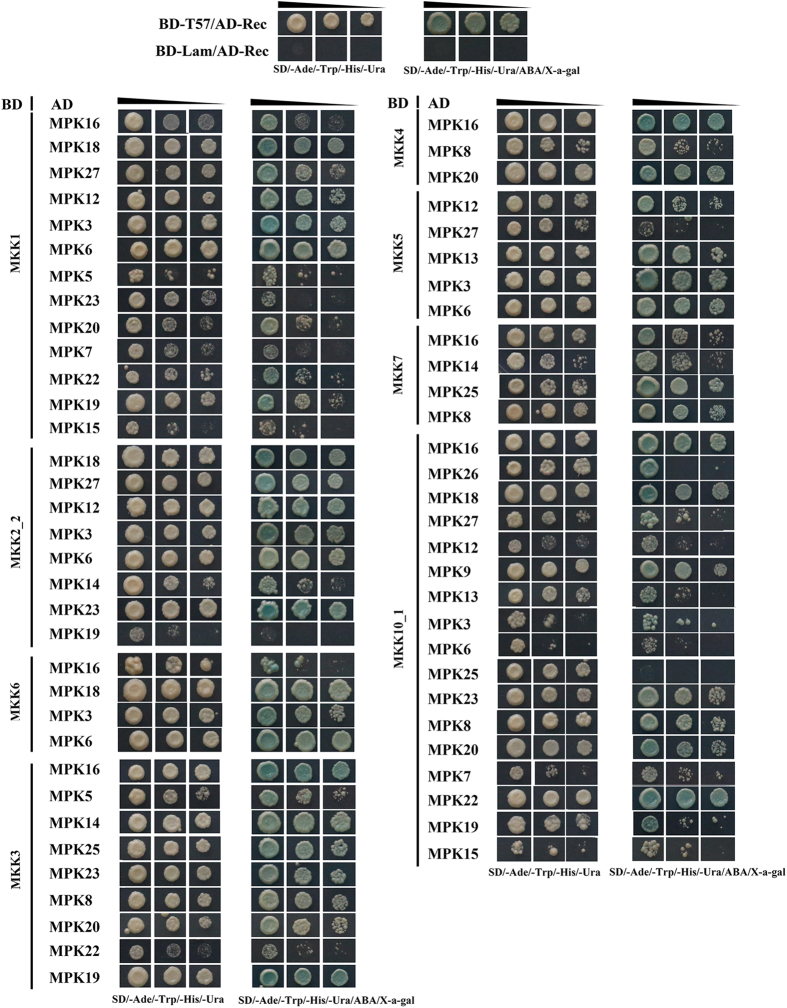
Comparative yeast two-hybrid interaction analyses of MKK with MAPKs. Interactions between selected MKKs and MAPKs. Yeasts harboring the indicated plasmid combinations were grown on selective medium SD/-Ade/-Trp/-His/-Ura, positive interactions were examined by addition of Aureobasidin A and X-α-gal. Positive (pGBKT7-53 + pGADT7-rec) and negative (pGBKT7-Lam+pGADT7-rec) controls. Repeated experiments showed similar results.

Each MKK-MAPK pair was individually co-transformed into yeast cells. Colonies were tested on quadruple dropout medium (SD/-Ade/-His/-Leu/-Trp) and subsequently on quadruple dropout medium supplemented with X-α-Gal and ABA. A total of 63 interactions were identified in the Y2H assay (Fig. 5). In the MKK-MAPK interaction pairs, eight of the MKK proteins were detected to interact with at least three MAPK proteins. GhMKK1, GhMKK2_2 and GhMKK6 in group A interacted with GhMPK3, GhMPK6 and GhMPK18. GhMKK1 and GhMKK2_2 also interacted with GhMPK12, GhMPK19, GhMPK23 and GhMPK27; and GhMKK1 and GhMKK6 interacted with GhMPK16. We also found that the genes that were duplicates of *GhMKK1*/*2_2* had different interactions. GhMKK1 interacted with GhMPK5, GhMPK7, GhMPK15, GhMPK16, GhMPK20 and GhMPK22, while its sister, GhMKK2_2, did not show any affinity to these proteins, instead, it was detected to interact with GhMPK14, indicating the functional divergence of paralogous genes during the evolutionary process. GhMKK3 interacted with the MAPKs in group C (GhMPK5, GhMPK8, GhMPK14, GhMPK20, GhMPK23, GhMPK25), and also interacted with GhMPK16, GhMPK19 and GhMPK22. Both GhMKK4 and GhMKK5 belong to group C, however, they interacted with different proteins. GhMKK4 interacted with GhMPK8, GhMPK16 and GhMPK20; while GhMKK5 interacted with GhMPK3, GhMPK6, GhMPK12, GhMPK13, and GhMPK27. GhMKK7 interacted with GhMPK8, GhMPK14, GhMPK16 and GhMPK25 (Fig. 5). Additionally, although expressed at a low level in all tissues analyzed, GhMKK10_1 was detected to interact with 17 of the 21 MAPK genes *in vitro*. It did not interact with GhMPK5, GhMPK10, GhMPK14 and GhMPK25 (data not shown), therefore its functional role needs to be further clarified.

**Figure 6:**
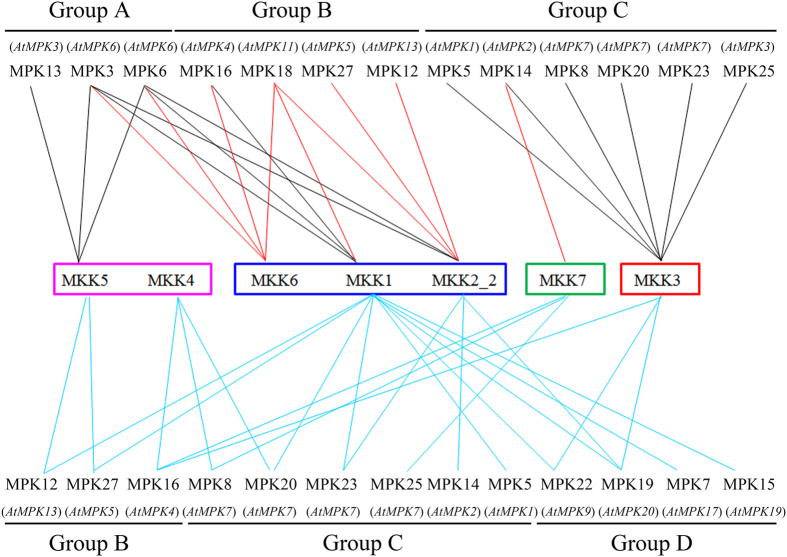
MKK-MAPK interaction in cotton compared to those in *Arabidopsis*. All the lines indicated the confirmed interactions of MKK-MAPK in cotton. Black lines indicated the interactions reported with confirmed function under stress in *Arabidopsis*, which was also identified in cotton. Red lines indicated the shared interactions in *Arabidopsis* and cotton, but no functions were reported in *Arabidopsis*. Blue lines indicated the interactions only confirmed in cotton without reported in *Arabidopsis*.

Of the 63 MKK-MAPK interactions detected, 23 have been reported in *Arabidopsis*, indicating that these interactions are reproducible in different species. The remaining 40 interaction pairs have not been reported in *Arabidopsis* and are novel interaction combinations found in cotton. We also found that each MKK protein was able to interact with at least three MAPK proteins, and multiple MKKs individually interacted with one MAPK, for example, GhMPK3 and GhMPK6 were able to interact with GhMKK1, GhMKK2_2, GhMKK5 and GhMKK6, indicating function universality in the MAPK signal pathway (Fig. 6).

### Comparison of the Expression Patterns Between MKK and MAPK Genes

The last components of the MAPK cascade (MAPK) in cotton development and responses to abiotic and biotic stress were characterized in our laboratory^36^. In the study, the majority of MKK genes in cotton were generally responsive to stress-related signals and abiotic stress treatments. Integrating these data, we compared the expression of MKKs and MAPKs in various tissues following stress-related signals and abiotic stress. In general, the expression levels of MKKs were lower than those of MAPKs in 22 *G. hirsutum* TM-1 tissues (Supplementary Fig. 6, Supplementary Dataset 3). Comparison of the expression of MKK and MAPK genes under stress-related signal and abiotic treatments revealed that MAPK genes were more sensitive to stress than MKK genes (Supplementary Figs 7–8, Supplementary Datasets 4–5). However, both MKK and MAPK genes were more sensitive to stress-related signals than abiotic stress.

**Figure 7:**
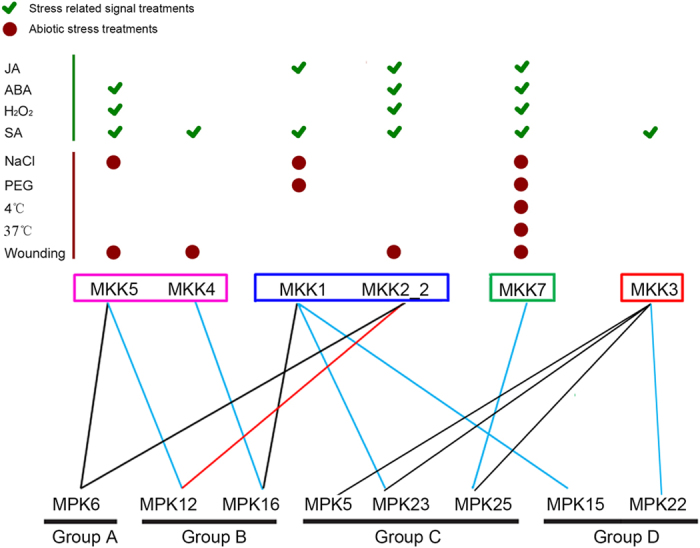
Integrated module of MKK-MAPK interactions in response to various treatments. The tick (green color) indicated the MKK-MAPK interactions commonly induced in stress-related signal treatments, including JA, ABA and SA. The circle (dark red) indicated the MPK-MPKK interactions commonly induced in stress treatment, including H_2_O_2_, NaCl, PEG, 4 °C, 37 °C and wounding. Black lines indicated the interactions reported with confirmed function under stress in *Arabidopsis*, which was also identified in cotton. Red lines indicated the shared interactions in *Arabidopsis* and cotton, but no functions were reported in *Arabidopsis*. Blue lines indicated the interactions newly confirmed in cotton without reported in *Arabidopsis*.

To further characterize the biological functions of the confirmed MKK-MAPK interaction pairs, we analyzed their responses to signal and stress treatments. Integration analysis of the interaction networks and expression patterns of MKK-MAPK family members revealed 13 potential MAPK signaling modules that are involved in the complicated cross-talk between hormones and abiotic stresses (Fig. 7). Genes involved in GhMKK1-GhMPK15/16/23 interactions were all induced by JA, SA, NaCl and PEG. In the GhMKK2_2-GhMPK6/12 modules, the transcript levels of *GhMKK2_2*, *GhMPK6* and *GhMPK12* were increased following JA, ABA, SA, H_2_O_2_ and wounding treatments. All of the GhMKK3-GhMPK5/22/23/25 interactions were induced by SA. Both genes involved in the GhMKK4-GhMPK16 interaction were induced by SA and wounding, and both genes involved in the GhMKK5-GhMPK6 interaction were induced by ABA, SA, H_2_O_2_, NaCl and wounding. In addition, in the GhMKK7-GhMPK25 module, *GhMKK7* and *GhMPK25* were induced by all tested signal-related and abiotic stresses. Taken together, these data highlight the important role of post-transcriptional regulation of MKK-MAPK signaling cascades and the complicated cross-talk between hormones and abiotic stresses.

## Discussion

### Characterization of MKKs in *G. Raimondii* and Their Evolution

Based on the genome scans of several plant genomes, MKK family genes have been systematically investigated in *Arabidopsis*^5^, *O. sativa*^41^, tomato^8^, maize^10^, cucumber^12^ and *B. distachyon*^13^. In the current study, a total of 11 MKKs from *G. raimondii* were identified and classified into four groups (A–D) according to their phylogenetic clades, which were similar to those reported previously^5^. The MKKs in groups B and C, MKK3, MKK4 and MKK5, were conserved in three species, *Arabidopsis*, *O. sativa* and *G. raimondii*. Group A and D MKK genes in *G. raimondii* were expanded in comparison with that in *Arabidopsis*, whereas MKK8 in group D in *Arabidopsis* was not detected in *G. raimondii*. This indicates that most MKK family members were conserved in dicots, while some members belonging to groups A and D were lost after the divergence of the monocots and dicots (Fig. 3). In addition, synteny analysis of the *G. raimondii* genome indicated that the MKK family mainly resulted from segmental duplication, which is consistent with the evolution of the MAPK family^36^.

### Functional Divergence of MKKs During Plant Growth and Development

Increasing evidence shows that MKK genes are involved in plant growth and development, and play key roles in the control of plant responses to abiotic stresses, biotic stresses and phytohormones^42^,^43^. For example, *AtMKK4* and *AtMKK5* control various aspects of stomatal development^44^, *AtMKK6* plays an important role in meiotic cytokinesis during pollen development^45^, *AtMKK7* has been shown to negatively regulate polar auxin transport^46^, *AtMKK9* plays an important role in leaf senescence^28^ and *OsMKK4* is a factor of seed/grain size and influences grain size in rice^47^. In the present study, we observed different expression patterns of MKKs in vegetative organs and reproductive organs in cotton. Two genes were constitutively expressed at high levels in both vegetative and reproductive organs. Two and three genes were expressed at high levels in vegetative organs and reproductive organs, respectively, and the remaining genes were expressed at low levels in all tested organs. The tissue- or organ-specific MKK expression patterns indicate their functional divergence during plant development and growth.

Accumulating evidence demonstrates that members of the MKK gene family in *Arabidopsis*, rice and other species function in response to abiotic and biotic stresses^3^. In *Arabidopsis*, *MKK1*/*2* plays important roles in the response to salt and cold stress and to pathogen attack^16^,^19^. *MKK4*/*5* plays a vital role in plant innate immunity^23^,^24^. *MKK3* is an essential component of ROS metabolism and is involved in JA and ABA stress signaling^20^,^22^,^48^,^49^. *MKK7* is involved in generating the mobile signal for SAR^50^. In cotton, *MKK1* contributes to the tolerance of salt and drought stress, and therefore is a crucial regulator of the response to environmental stresses^30^. *MKK4* participates in regulating abscisic acid, gibberellin and hydrogen peroxide signaling^32^. *MKK5* plays a negative role in the response to salt and drought stresses^33^. In our study, all 10 detected MKKs showed differential expression patterns in response to stress-related signals and abiotic stresses (Fig. 4). All MKK genes were induced by at least one stress-related signal treatment. 40% of the MKK genes were induced by three stress-related signals, and 20% was induced by two stress-related signals. The remaining genes were responsive to SA. Furthermore, *GhMKK7* was responsive to six abiotic stresses, *GhMKK6* was responsive to five abiotic stresses, *GhMKK5* was responsive to three abiotic stresses, and 20% of MKK genes were responsive to four and two abiotic stresses, respectively. 20% were responsive to one of the abiotic stresses tested. In addition, *GhMKK3* was insensitive or down-regulated in response to abiotic stresses. Taken together, these results show that MKKs are generally responsive to stress-related signals and abiotic stress treatments, and play an important role in the responses to environmental stresses. This highlights the extent of cross-talk that exists between stress-related signals and abiotic stress.

### Integration analysis of the interaction levels and expression patterns of MKKs and MAPKs

Plant MAPKs regulate numerous physiological responses by interacting with upstream and downstream protein components. To date, the most extensively characterized MKK-MAPK interaction partners are from *Arabidopsis*^51^, *O. sativa*^6^,^52^, maize^10^, canola^53^, watermelon^54^ and *B. distachyon*^13^, and these have elucidated the cross-talk between different signaling pathways. Reports on MKK-MAPK interactions in cotton are limited. Here, we systematically analyzed interactions between eight MKKs and 21 MAPKs using the Y2H system. In addition to those of GhMKK10_1, a total of 46 interactions were identified, as shown in Fig. 6. We found 23 MKK-MAPK interactions that were consistent with those reported previously in *Arabidopsis*, and the function of 14 of these MKK-MAPK interaction pairs has been elucidated. In *Arabidopsis*, MKK1/2-MPK4/6 cascades have previously been shown to play important roles in the response to salt and cold stress and to pathogen attack^18^,^19^, which is consistent with our observation that GhMKK1/2_2 significantly interacted with GhMPK3/6, and that GhMKK1 interacted with GhMPK16, and the orthologous of relationship are as shown in Fig. 6. AtMKK3 has been reported to act on AtMPK7 and AtMPK8 to mediate ROS signaling and to regulate AtMPK6 in response to JA^20^,^21^. In addition, AtMKK3 plays a role in ABA stress signaling, which subsequently stimulates the activity of the group C MAPKs, including AtMPK1, AtMPK2, AtMPK7 and AtMPK14^22^. We also found that GhMKK3 significantly interacted with group C MAPKs, which including GhMPK5, GhMPK8, GhMPK14, GhMPK20, GhMPK23 and GhMPK25. AtMEKK1-AtMKK4/5-AtMPK3/6 cascades play vital roles in the plant innate immunity^23^,^24^. We also revealed that GhMKK5 significantly interacted with GhMPK13, GhMPK3 and GhMPK6. The interacting pairs and their associated complicated linear MAPK pathways function in regulating plant immune responses and other processes in cotton and are worth analyzing further. There were nine other interaction patterns (red lines in Fig. 6), consistent with *Arabidopsis* homologs: GhMKK1 interacted with GhMPK18; GhMKK2_2 interacted with GhMPK12, GhMPK18 and GhMPK27; GhMKK6 interacted with GhMPK3, GhMPK6, GhMPK16 and GhMPK18; and GhMKK7 interacted with GhMPK14. However, no function was reported for any of these interactions, therefore their functional roles are also worth clarifying further. In addition, 23 interactions differed from the previous observations in *Arabidopsis*. For example, GhMKK1 interacted with GhMPK5, GhMPK7, GhMPK12, GhMPK15, GhMPK19, GhMPK20, GhMPK22, GhMPK23 and GhMPK27; GhMKK2_2 interacted with GhMPK14, GhMPK19 and GhMPK23; GhMKK3 interacted with GhMPK16, GhMPK19 and GhMPK22; GhMKK4 interacted with GhMPK8, GhMPK16 and GhMPK20; GhMKK5 interacted with GhMPK12 and GhMPK27; and GhMKK7 interacted with GhMPK8, GhMPK16 and GhMPK25 (Fig. 6). These new interaction pairs in cotton imply an inherent difference in signaling pathways between *Arabidopsis* and cotton, and highlight the necessity to explore the MAPK-mediated signaling pathways for use in genetic investigations and breeding in cotton.

MAPK modules are known to be involved in various stress-related signals and abiotic stress responses. In *Arabidopsis*, *MKK1/2* has previously been shown to play important roles in the response to salt and cold stress^18^,^19^. In our study, the GhMKK1-GhMPK15/16/23 interactions were induced by JA, SA, NaCl and PEG. *GhMKK1* was found to play an important role in the tolerance of salt, drought and pathogen attack, and *GhMPK7* (a paralog of *GhMPK23*) has a role in broad spectrum disease resistance^55^, suggesting that the GhMKK1-GhMPK15/16/23 interactions might be involved in salt and drought stress as well as defense against pathogens. All of the GhMKK2_2-GhMPK6/12 modules were induced JA, ABA, SA, H_2_O_2_ and wounding, and silencing of *GhMKK2* compromises the resistance to cotton pathogens^31^, implying that GhMKK2_2-GhMPK6/12 plays an important role in the response to pathogen attack. *AtMKK3* is an essential component of ROS metabolism and is involved in JA and ABA stress signaling^20^,^21^,^22^. In our study, GhMKK3-GhMPK5/22/23 interactions were induced by SA, suggesting that this MAPK cascade may be involved in generating the mobile signal for SAR in cotton. MKK4/5-MPK3/6 cascades play vital roles in innate immunity in *Arabidopsis*^23^,^24^. However, this is not the case for *MKK4* and *MKK5* in cotton; *GhMKK4* plays an important role in abscisic acid, gibberellin and hydrogen peroxide signaling^32^. *GhMKK5* can be induced by pathogen infection, abiotic stresses and multiple defense-related signal molecules^33^, implying that MKK4 and MKK5 may have different interaction substrates, and their functions are slightly different^32^,^33^. In our study, GhMKK4 and GhMKK5 both belonged to group C, but they interacted with different MAPKs. In the GhMKK4-GhMPK16 interaction, both genes were induced by SA and wounding, indicating that they are likely involved in the response to wounding stress and in SA signaling pathways. In the GhMKK5-GhMPK6 interaction, both genes were induced by ABA, SA, H_2_O_2_, NaCl and wounding, which suggests that the interaction might be involved in multiple signaling pathways and the response to salt stress. *AtMKK7* has been shown to negatively regulate polar auxin transport and is involved in generating the mobile signal for SAR^46^,^50^. In the GhMKK7-GhMPK25 module, *GhMKK7* and *GhMPK25* were induced by all signal-related stresses and abiotic stresses. *GhMPK7* (a paralog of *GhMPK25*) has a role in broad spectrum disease resistance^55^, and we also demonstrated that *GhMPK25* is an important component of cotton resistance to *V. dahliae*^36^, which suggests that GhMKK7-GhMPK25 might be involved in multiple signaling pathways and responses to stress.

In summary, we performed the first comprehensive investigation of the MKK gene family in cotton involving in the analysis of sequence phylogeny, genomic structure, chromosomal location and adaptive evolution. The temporal and spatial expression patterns in vegetative and reproductive organs in response to stress-related signals and abiotic stresses revealed that MKKs may play a crucial role in driving evolutionary novelty and adaptation to new environments. Genome-wide MKK-MAPK interaction pairs were systematically identified in cotton, and 40 new interaction pairs were identified. Combining MKK-MAPK interaction pairs with their expression patterns further revealed 13 potential MAPK signaling modules involved in the complicated cross-talk between hormones and abiotic stresses. Our study is the first to systematically elucidate the relationship of the last two kinases of the MAPK cascade in different organs and under different stress conditions and this provides an important foundation for the further functional dissection of the last two kinases of the MAPK cascade for utilization in cotton breeding.

## Methods

### Characterization of MKK Genes in *G. Raimondii*

The MKKs in *Arabidopsis* and *O. sativa* were used as queries to search for putative MKK proteins against the *G. raimondii* genome^37^ database at http://www.phytozome.net. The PFAM (http://pfam.sanger.ac.uk) and SMART (http://smart.emblheidelberg.de/) databases were employed to detect conserved domains. The isoelectric points and molecular weights of the MKK proteins were predicted on the ExPASy proteomics server (http://expasy.org/). The subcellular localization of each MKK was analyzed using the CELLO v2.5 server (http://cello.life.nctu.edu.tw/). Sequence alignment was carried out by the Clustal X program and a phylogenetic tree was constructed by the Maximum likelihood (ML) method in MEGA6. Mapping of MKK genes was performed using MapInspect (http://www.plantbreeding.wur.nl/UK/software_mapinspect.html). Segment duplicates were identified using Plant Genome Duplication Database (http://chibba,agtec.uga.edu/duplication), tandem duplicates were defined as genes separated by five or fewer genes. The exon/intron structures of individual MKK genes were determined by aligning the cDNA sequences to their corresponding genomic DNA sequences.

### Transcriptome Data of the MKK and MAPK Family Genes in *G. Hirsutum*

The RNA-Seq data from distinct tissues have been previously reported in TM-1 genome sequencing research^38^, which was downloaded from http://mascotton.njau.edu.cn and NCBI database SRA: PRJNA248163. All these tissues include the root, stem and leaves of two week-old plants; torus, petal, stamen, pistil and calycle dissected from whole mature flowers; fiber bearing ovule at −3, −1, 0, 1, 3 DPA, ovules at 5, 10, 20, 25 and 35 DPA, and fibers at 5, 10, 20 and 25 DPA. The fragments per kilobase of exon per million fragments (FPKM) was used to estimate the gene expression level of MKK and MAPK family genes in distinct tissue of *G. hirsutum*.

### Plant materials and Treatments

*G. hirsutum* L. cv. Jinmain 19, which exhibits a high tolerance to abiotic stress, was used for the abiotic stress treatments. Cotton seedlings (*G. hirsutum* L.cv. Jinmian19) were grown in a growth chamber under greenhouse conditions at 28 °C under a 16 h light/8 h dark cycle. Three-week-old cotton seedlings were used for the following treatments. For signaling substance treatments, leaves were sprayed with 100 μM JA, 100 μM ABA or 10 mM SA (ddH_2_O as a solvent control). For oxidative stress, leaves were sprayed with 10 mM H_2_O_2_ (ddH_2_O as a solvent control). For the salt and drought treatments, the roots of cotton seedlings were irrigated with 200 mM NaCl and 20% PEG, respectively (ddH_2_ O as a mock control). For temperature stress treatments, the seedlings were placed in a growth chamber at a high temperature (37 °C) or a low temperature (4 °C) (28 °C as a mock control). Seedling leaves were cut with scissors for wound treatment. The leaves were harvested at the appropriate time points (triplicate samples were collected at each time point [n = 3 seedlings]). All tissues were flash-frozen in liquid nitrogen and stored at −70 °C for further analysis.

### Extraction of Total RNA

Cotton total RNA was extracted from leaves and roots using a Biospin plant Total RNA extraction kit (BioFlux) according to the manufacturer’s protocol. RNA was then treated with Dnase I (Invitrogen, http://www.invitrogen.com) to remove genomic DNA. Purified RNA (2 μg) was subsequently reverse transcribed using a first-strand cDNA synthesis kit according to the manufacturer’s instructions. The primer pairs used for real-time PCR were designed using Beacon Designer 7.0 according to cotton MKK gene sequences and synthesized commercially (Supplementary Dataset 6). The His3 (AF024716) gene was used as a control. Real-time PCR was performed on an ABI7500 Real time PCR system (Applied Biosystems, USA) using SYBR Green (Roche, Switzerland) with three replicates. The PCR program was as follows: initial denaturation at 95 °C for 10 min, 40 cycles of denaturation at 95 °C for 15 s, 60 °C for 15 s and 72 °C for 15 s. The expression levels of MKK genes were calculated according to Livak and Schmittgen^56^.

### Yeast Two-hybrid Assays

Putative interactions between MKKs and MAPKs were examined using the matchmaker Gold Yeast Two-Hybrid system according to the manufacturer’s instructions (Clontech, Mountain View, CA, USA). The coding sequences of MKKs and MAPKs were amplified using gene-specific primers from *G. hirsutum* acc. TM-1 and cloned into pGBKT7 and pGADT7 vectors (Supplementary Dataset 6). Eight MKK proteins were transformed into Y2H Gold yeast strains and the auto-activation activity was tested. Aureobasidin A (ABA) was used to inhibit the auto-activation^57^. Individual MKK-MAPK pairs were co-transformed into yeast cells, and the resulting colonies were tested on selective medium (SD/-Trp/-Leu and SD/-Trp/-Leu/ABA), then on quadruple dropout medium (SD/-Ade/-His/-Leu/-Trp) and quadruple dropout medium supplemented with X-α-Gal and Aureobasidin A for 7days at 30 °C, only yeast colonies with interactions between MKKs and MAPKs were able to grow on the selection media. Co-transformation of pGBKT7-53, and pGBKT7-Lam and pGADT7-rec, were as positive and negative controls, respectively.

### Statistical Analysis

All experiments were repeated independently at least three times. Data obtained were subjected to statistical analysis using student’s t-tests and probability values of P < 0.05 were considered as significant between the different treatments.

## Additional Information

**How to cite this article**: Zhang, X. *et al*. Integration analysis of MKK and MAPK family members highlights potential MAPK signaling modules in cotton. *Sci. Rep*. **6**, 29781; doi: 10.1038/srep29781 (2016).

## Supplementary Material

Supplementary Information

Supplementary Dataset S1

Supplementary Dataset S2

Supplementary Dataset S3

Supplementary Dataset S4

Supplementary Dataset S5

Supplementary Dataset S6

## Figures and Tables

**Table 1:** Genome-wide analysis of MKK genes in *G. raimondii*.

Name	ID*	Chr**	Published MKK	Accession number	AA length	pI	MW(Kd)	Group	Subcellular localization***
GrMKK1	Gorai.009G033800.1	D05(Chr09)	MKK1(HQ828075.1)	KX118695	361	5.49	40.09	A	Cytoplasmic
GrMKK2_1	Gorai.001G013600.1	D07(Chr01)			362	6.19	40.23	A	Out membrane
GrMKK2_2	Gorai.010G085300.1	D06(Chr10)		KX118696	364	8.71	40.91	A	Out membrane
GrMKK6	Gorai.013G229600.1	D13(Chr13)		KX118697	354	6.28	39.93	A	Outmembrane
GrMKK3	Gorai.007G075600.1	D11(Chr07)		KX118698	518	5.62	57.65	B	Cytoplasmic
GrMKK4	Gorai.010G221800.1	D06(Chr10)	MKK4(FJ966866)	KX118699	349	9.3	38.88	C	Periplasmic
GrMKK5	Gorai.009G117800.1	D05(Chr09)	MKK5(HQ637469.1)	KX118700	350	8.92	38.91	C	Periplasmic
GrMKK7	Gorai.008G291700.1	D12(Chr08)		KX118701	323	8.4	36.35	D	Cytoplasmic
GrMKK9	Gorai.008G291800.1	D12(Chr08)	MKK9(HM989878.1)		321	7.63	36.23	D	Cytoplasmic
GrMKK10_1	Gorai.003G184000.1	D03(chr03)		KX118702	322	7.1	35.74	D	Cytoplasmic
GrMKK10_2	Gorai.008G225100.1	D12(Chr08)			320	7.09	35.42	D	Cytoplasmic

^*^Sequence information from *G. raimondii*^37^.

^**^Chromosome numbers D1 to D13 refer to chromosomes name of D subgenome in allotetraploid cultivated cotton species^38^, and the names of 13 scaffolds from the *G. raimondii* genome^37^ are shown in brackets.

^***^Subcellular localization predicted using software (http://cello.life.nctu.edu.tw).

**Table 2:** Expression profiles of MKK genes under different stress treatments in cotton.

Gene	Group	Signaling molecules			Environmental stress factors					
		JA (100 μM)	ABA (100 μM)	SA (10 mM)	H_2_O_2_ (10 mM)	Salt (200 mM)	PEG6000 (20%)	4 °C	37 °C	Wounding
MKK1	A	**	*	**	*	**	**	–	–	*
MKK2_1	A	*	*	**	*	**	*	–	–	*
MKK2_2	A	**	**	**	**	–	*	D	D	**
MKK6	A	**	**	**	**	*	**	**	**	**
MKK3	B	*	D	**	*	D	–	D	D	D
MKK4	C	–	*	**	D	*	–	–	–	**
MKK5	C	–	**	**	**	**	*	*	*	**
MKK7	D	**	**	**	**	**	**	**	**	**
MKK10_1	D	**	**	**	**	**	*	**	D	**
MKK10_2	D	*	–	**	**	**	D	**	D	**

Note: For hormone treatments, the leaves of seedlings were harvested at 0, 0.5, 1, 2, 4, 6, 8, 10, 12 and 24 h after treatment; For the environmental stress factor treatments, the leaves of seedlings were harvested at 0, 0.5, 1, 2, 4, 6, 8, 10, 12 and 24 h after treatment; “**” and “*” indicate significant difference at P < 0.01 and P < 0.05, respectively; “–” represents no change and weak upregulation; “D” represents significant reduction in MKK gene expression after treatment; The Student’s t-test was performed between treated samples and untreated samples.
