# Supplementary material for: Integration analysis of MKK and MAPK family members highlights potential MAPK signaling modules in cotton
Source: Sci Rep. 2016 Jul 15;6:29781. doi: 10.1038/srep29781 (PMC4945917; doi:10.1038/srep29781)
Supplement: Supplementary Information [file srep29781-s1.pdf]

Integration analysis of MKK and MAPK family members highlights potential MAPK signaling modules in cotton

Xueying Zhang, Xiaoyang Xu, Yujia Yu, Chuan Chen, Jing Wang, Caiping Cai, Wangzhen Guo*

State Key Laboratory of Crop Genetics & Germplasm Enhancement, Hybrid Cotton R & D Engineering Research Center, Ministry of Education, Nanjing Agricultural University, Nanjing 210095, China

*** Correspondence and requests for materials should be addressed to W.Z.G (email: moelab@njau.edu.cn)**

Supporting Information

Supplementary Table 1. Pearson correlation for the expression profiles of paralogous pairs.

Supplementary Table 2. Comparison on the nucleotide and amino sequence of MKK and MAPK homoeologs in A- and D-subgenome.

Supplementary Figure 1. Conserved motif and domains of MKKs in *G. raimondii*.

Supplementary Figure 2. Expression patterns of MKK genes in different tissues and organs in *G. hirsutum* TM-1.

Supplementary Figure 3. Expression patterns of MKK genes under stress-related signal treatments (JA, ABA and SA).

Supplementary Figure 4. Expression patterns of MKK genes under stress treatments (H_2O_2, NaCl, PEG, 4℃, 37℃ and wounding).

Supplementary Figure 5. Detection of the transcriptional activation activity of MKK bait vectors.

Supplementary Figure 6. Expression distribution of MKK and MAPK genes across 22 tissues in *G. hirsutum* TM-1.

Supplementary Figure 7. Comparison of expression level of MKK and MAPK genes under different stress-related signal treatments.

Supplementary Figure 8. Comparison of the expression level of MKK and MAPK genes under different stress treatments.

Supplementary Table 1. Pearson correlation for the expression profiles of paralogous pairs.

Gene1	Gene2	Similarity	Correlation* coefficient in organs	Correlation coefficient in different hormones			Correlation coefficient in different stresses					
				JA	ABA	SA	H_2O_2	NaCl	PEG	4℃	37℃	Wounding
MKK1	MKK2_1	85.00%	-0.1009	-0.4566	**0.6191**	**0.9595**	**0.6639**	0.3836	0.4004	**0.8401**	**0.5547**	**0.6047**
MKK1	MKK2_2	87.00%	-0.1434	-0.5324	**0.7859**	**0.9729**	**0.9071**	**0.8426**	**0.7638**	**0.7359**	**0.8697**	**0.8445**
MKK2_1	MKK2_2	90.00%	**-0.0817**	**0.7399**	**0.5839**	**0.9921**	**0.8336**	**0.5871**	**0.6806**	**0.6185**	**0.7378**	**0.8096**
MKK10_1	MKK10_2	85.00%	0.1271	0.1186	**0.7262**	**0.6643**	**0.5258**	**0.5518**	-0.1653	0.1425	**0.7218**	-0.1739

Note: Correlation of expression pattern between Gene1 and Gene2 in organs is calculated using expression levels of genes in different hormones and different stresses. *correlation coefficient: r>0.5: positive correlation; 0<r<0.5: no clear positive correlation; -0.5<r<0: no clear negative correlation; r<-0.5: negative correlation

Supplementary Table 2. Comparison on the nucleotide and amino sequence of MKK and MAPK homoeologs in A- and D-subgenome.

Name	A-subgenome	D-subgenome	Identified	
			CDS	AA
MKK	GhMKK1A	GhMKK1D	98%	98%
	GhMKK2_2A	GhMKK2_2D	98%	97%
	GhMKK3A	GhMKK3D	99%	99%
		GhMKK4D	/	/
	GhMKK5A	GhMKK5D	98%	99%
	GhMKK6A	GhMKK6D	99%	99%
	GhMKK7A	GhMKK7D	98%	98%
	GhMKK10_1A	GhMKK10_1D	98%	99%
MAPK	GhMAPK3A	GhMAPK3D	98%	99%
	GhMAPK5A	GhMAPK5D	98%	99%
	GhMAPK6A	GhMAPK6D	99%	99%
	GhMAPK7A	GhMAPK7D	97%	98%
	GhMAPK8A	GhMAPK8D	99%	100%
	GhMAPK9A	GhMAPK9D	98%	99%
	GhMAPK10A	GhMAPK10D	99%	99%
	GhMAPK12A	GhMAPK12D	98%	99%
	GhMAPK13A	GhMAPK13D	99%	99%
	GhMAPK14A	GhMAPK14D	99%	99%
	GhMAPK15A	GhMAPK15D	98%	98%
	GhMAPK16A	GhMAPK16D	98%	100%
	GhMAPK18A	GhMAPK18D	98%	97%
		GhMAPK19D	/	/
	GhMAPK20A	GhMAPK20D	99%	99%
	GhMAPK22A	GhMAPK22D	99%	99%
	GhMAPK23A	GhMAPK23D	98%	99%
	GhMAPK25A	GhMAPK25D	98%	99%
	GhMAPK26A	GhMAPK26D	97%	97%
	GhMAPK27A	GhMAPK27D	99%	100%
	GhMAPK28A	GhMAPK28D	98%	99%

Supplementary Figures

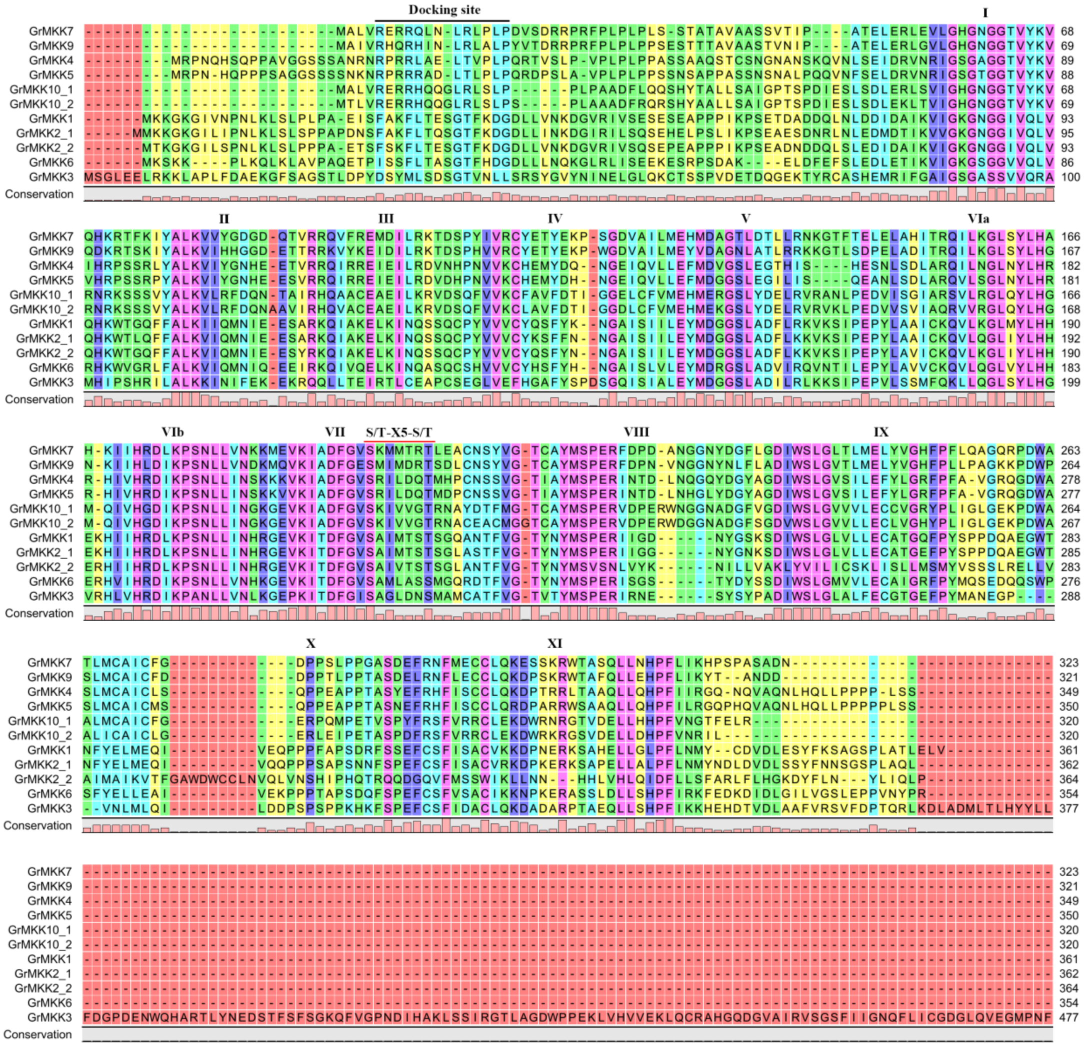

Supplementary Figure 1. Conserved motif and domains of MKKs in *G. raimondii*.

Roman numerals indicate regions containing the 11 domains (I-XI) found in the cotton PK subdomains. The conserved consensus motif, S/T-X5-S/T between subdomains VII and VIII of the MKKs are highlighted with red line.

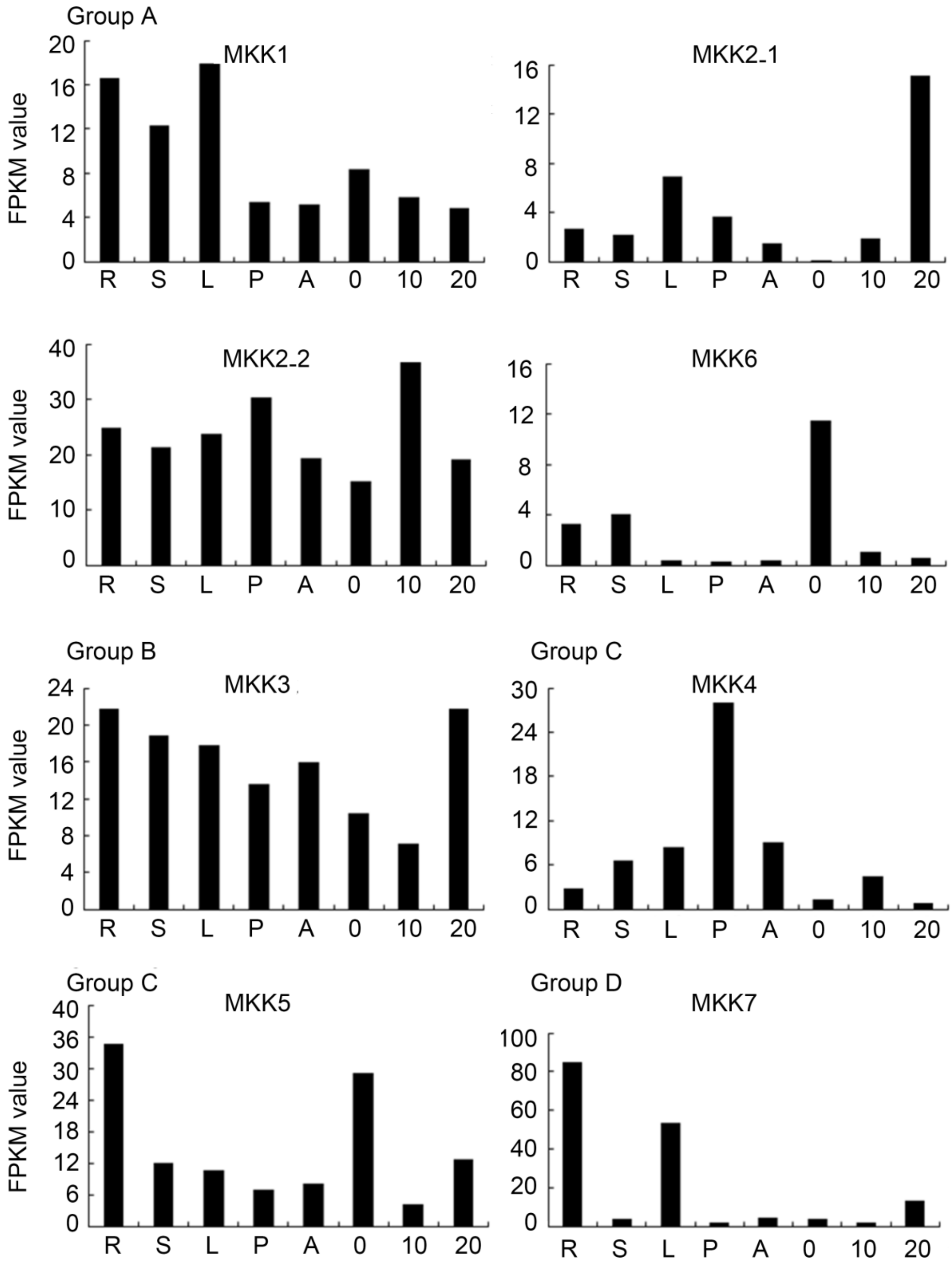

Supplementary Figure 2. Expression patterns of MKK genes in different tissues and organs in *G. hirsutum* TM-1.

Transcriptome level of MKKs in eight cotton tissues (Root; Stem; Leaf; Petal; Anther; Ovule at 0 dpa; Fiber at 10 dpa; and Fiber at 20dpa) were obtained with fragments per kilobase of exon per million fragments (FPKM) values.

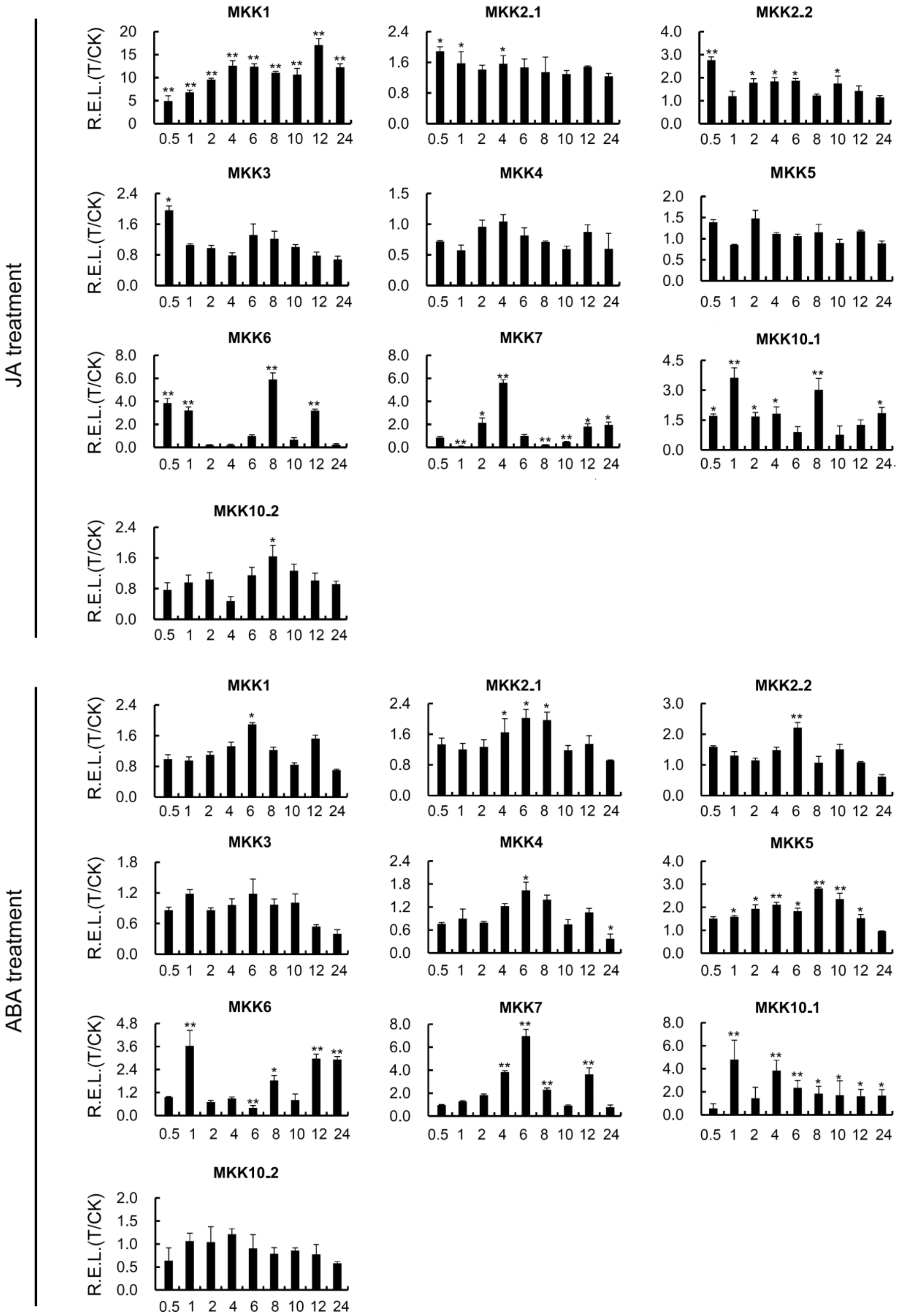

Supplementary Figure 3. Expression patterns of MKK genes under stress-related signal treatments (JA, ABA and SA). (Continue to next page)

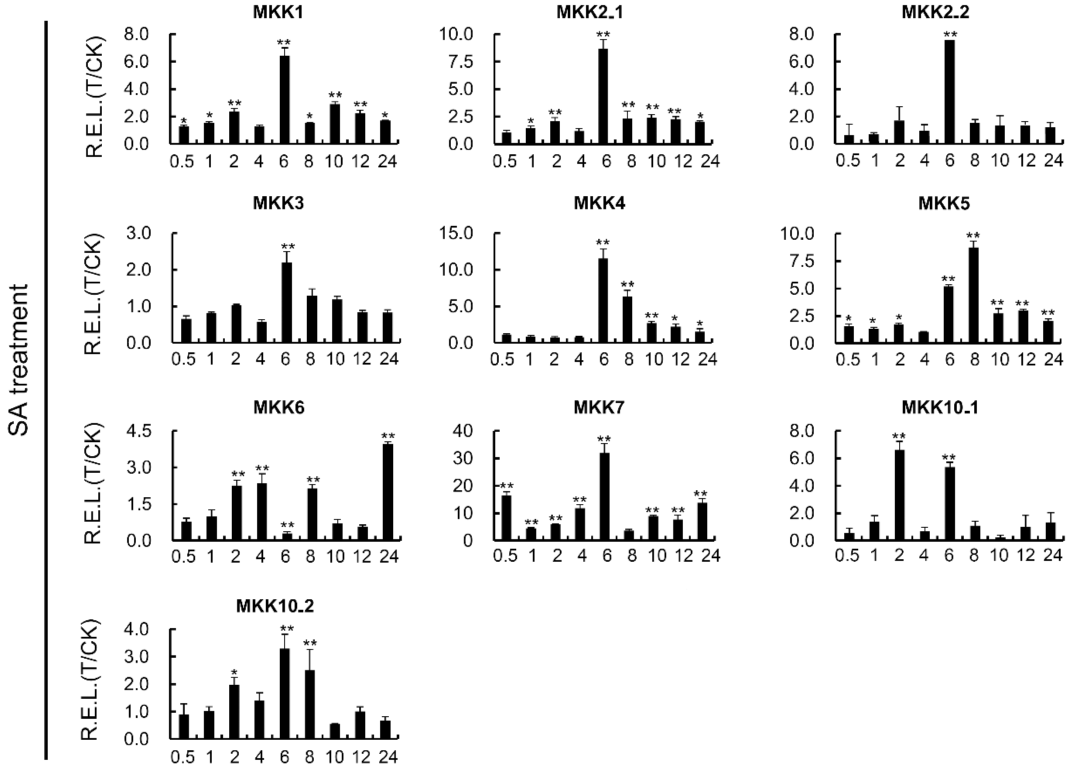

Supplementary Figure 3. Expression patterns of MKK genes under stress-related signal treatments (JA, ABA and SA).

The expression patterns of MKK genes were presented as the ratio of relative expression levels (R.E.L.) in stress-signal treatments (T) and the corresponding controls (CK). The X-axis indicates the hours of stress-related signal treatments and the Y-axis indicates T/CK value. The error bars were calculated based on three biological replicates using standard deviation. "*": significant difference ($p<0.05$); "**": significant difference ($p<0.01$).

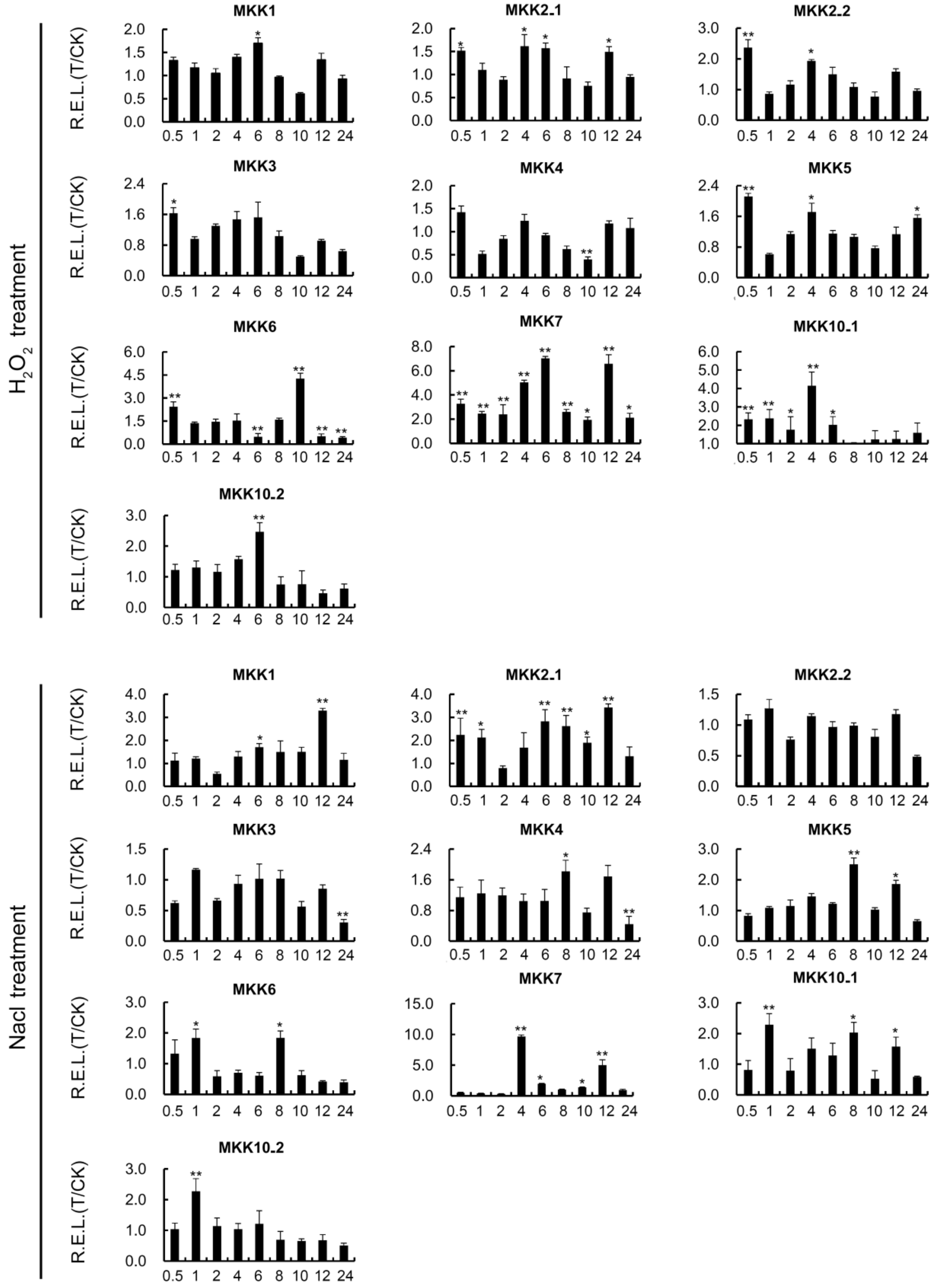

Supplementary Figure 4. Expression patterns of MKK genes under stress treatments (H_2O_2, NaCl, PEG, 4℃, 37℃ and wounding). (Continue to next page)

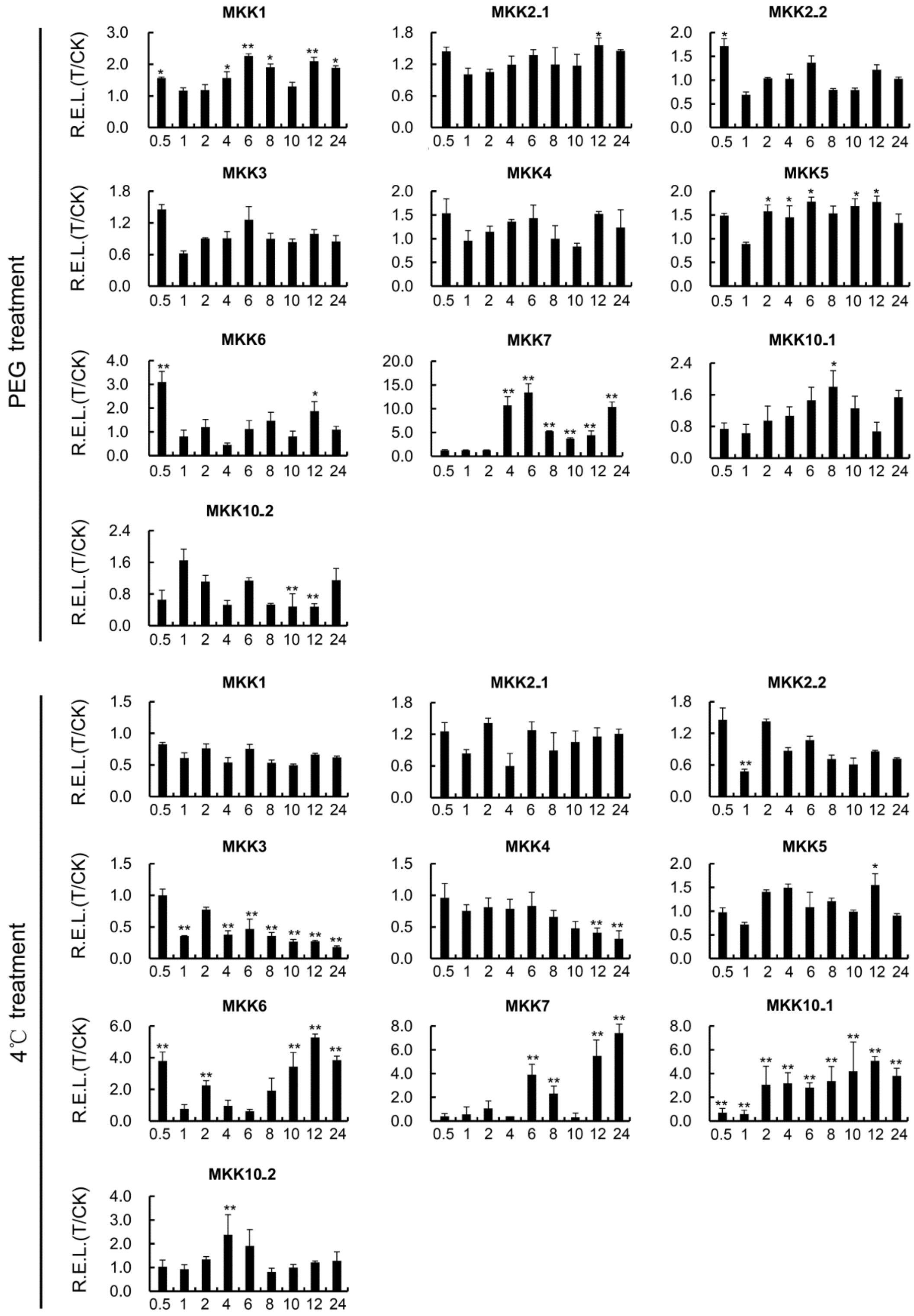

Supplementary Figure 4. Expression patterns of MKK genes under stress treatments (H_2O_2, NaCl, PEG, 4℃, 37℃ and wounding). (Continue to next page)

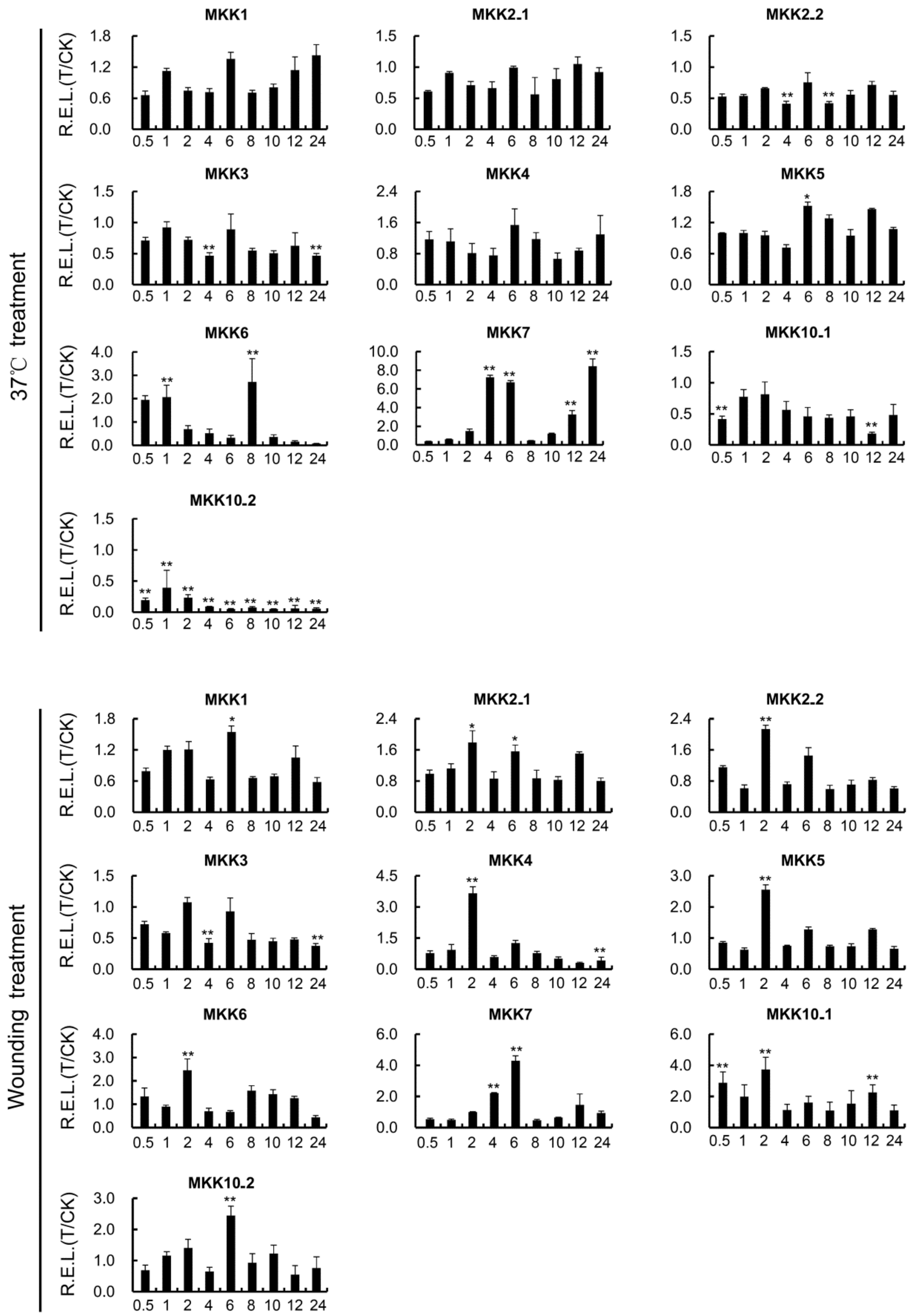

Supplementary Figure 4. Expression patterns of MKK genes under stress treatments (H_2O_2, NaCl, PEG, 4℃, 37℃ and wounding).

The expression patterns of MKK genes were presented as the ratio of relative expression levels (R.E.L.) in stress-signal treatments (T) and the corresponding controls (CK). The

X-axis indicates the hours of stress-related signal treatments and the Y-axis indicates T/CK value. The error bars were calculated based on three biological replicates using standard deviation. “*”: significant difference ($p<0.05$); “**”: significant difference ($p<0.01$).

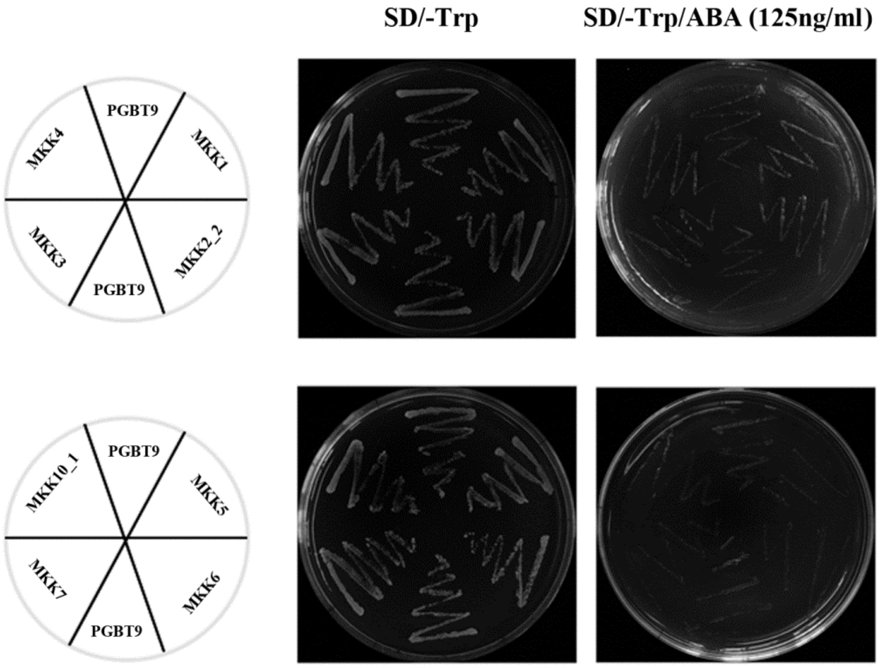

Supplementary Figure 5. Detection of the transcriptional activation activity of MKK bait vectors.

Yeasts harboring the indicated plasmid combinations were grown on selective medium SD/-Trp, positive interactions were examined by addition of Aureobasidin A (ABA).

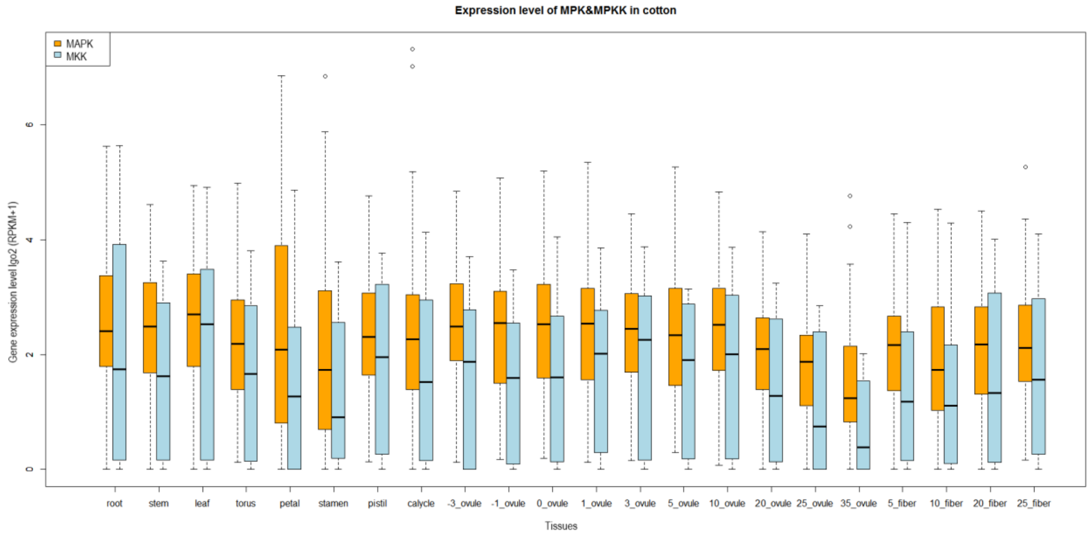

Supplementary Figure 6. Expression distribution of MKK and MAPK genes across 22 tissues in *G. hirsutum* TM-1.

The log_2FPKM values of genes in each tissue were showed as a boxplot. Yellow and blue box indicated the MKK and MAPK genes, respectively. The black line in the each box was the median of the data set.

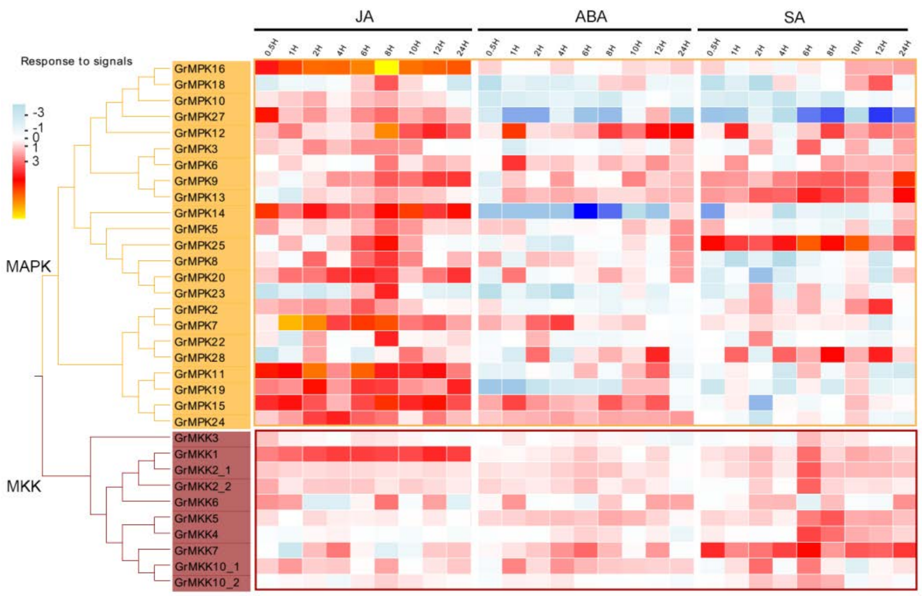

Supplementary Figure 7. Comparison of expression level of MKK and MAPK genes under different stress-related signal treatments.

The data were presented according to the phylogenetic tree using $Log_2^{T/C}$ (fold-changes) of relative expression level of all genes in response to stress-signal treatments (Treatments), in comparison to treatment with H_2O as mock control (Control). Red and light blue colors represented the up- and down-regulated expression level under treatment. The stress-related signals were involved in JA, ABA and SA, respectively.

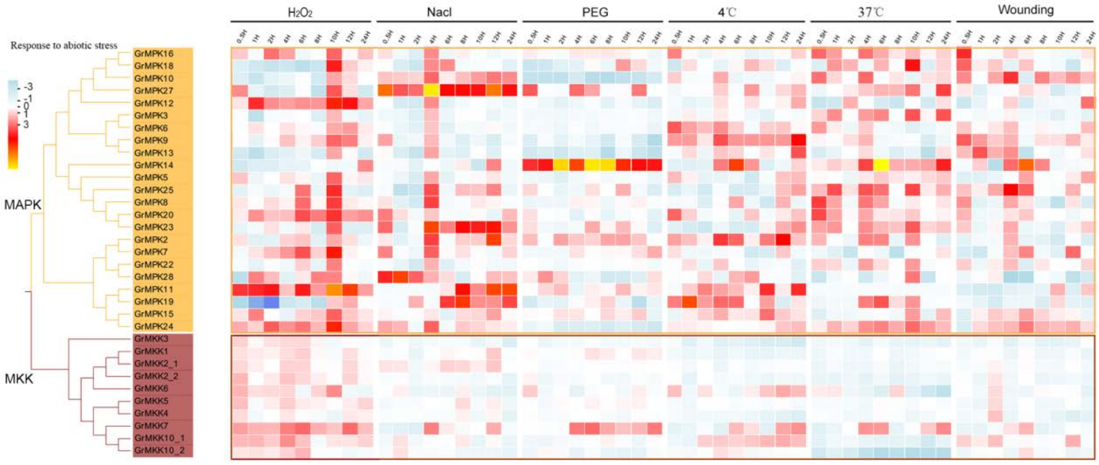

Supplementary Figure 8. Comparison of the expression level of MKK and MAPK genes under different stress treatments.

The data are presented in clusters using the fold-change ($Log_2^{T/C}$) of relative expression for all genes in response to abiotic stress treatments (Treatments), in comparison to their respective controls (Control). Red and blue colors represent the increased and decreased expression levels under different stresses, respectively, in comparison to controls. The abiotic stresses were involved in H_2O_2, NaCl, PEG, 4℃, 37℃, and wounding, respectively.
